# TOE1/TOE2 Interacting with GIS to Control Trichome Development in *Arabidopsis*

**DOI:** 10.3390/ijms24076698

**Published:** 2023-04-03

**Authors:** Yihua Liu, Shuaiqi Yang, Ali Raza Khan, Yinbo Gan

**Affiliations:** 1 College of Agriculture and Forestry Sciences, Linyi University, Linyi 276000, China; 2 Zhejiang Key Lab of Crop Germplasm, Department of Agronomy, College of Agriculture and Biotechnology, Zhejiang University, Hangzhou 310027, China

**Keywords:** trichome, TOE1, GIS, protein interaction, *Arabidopsis*

## Abstract

Trichomes are common appendages originating and projecting from the epidermal cell layer of most terrestrial plants. They act as a first line of defense and protect plants against different types of adverse environmental factors. GL3/EGL3-GL1-TTG1 transcriptional activator complex and GIS family genes regulate trichome initiation through gibberellin (GA) signaling in *Arabidopsis.* Here, our novel findings show that TOE1/TOE2, which are involved in developmental timing, control the initiation of the main-stem inflorescence trichome in *Arabidopsis.* Phenotype analysis showed that the *35S:TOE1* transgenic line increases trichome density of the main-stem inflorescence in *Arabidopsis*, while *35S:miR172b*, *toe1*, *toe2* and *toe1toe2* have the opposite phenotypes. Quantitative RT-PCR results showed that *TOE1*/*TOE2* positively regulate the expression of *GL3* and *GL1.* In addition, protein-protein interaction analysis experiments further demonstrated that TOE1/TOE2 interacting with GIS/GIS2/ZFP8 regulate trichome initiation in *Arabidopsis*. Furthermore, phenotype and expression analysis also demonstrated that TOE1 is involved in GA signaling to control trichome initiation in *Arabidopsis*. Taken together, our results suggest that TOE1/TOE2 interact with GIS to control trichome development in *Arabidopsis.* This report could provide valuable information for further study of the interaction of TOE1/TOE2 with GIS in controlling trichome development in plants.

## 1. Introduction

Trichomes are a common structure on the upper side of most terrestrial plants. They have different shapes and sizes and are often used as an important basis for plant species identification [1,2,3,4]. On the basis of their diversity and ease of access and observation, trichomes have become an excellent molecular model for studying cell-differentiation processes such as fate determination, cell-cycle control and cell morphogenesis in plants [5,6]. As the first layer of the physical protection barrier, trichomes can effectively protect plants from mechanical damage, ultraviolet radiation, pest erosion and water loss via transpiration and help maintain the normal growth of plants [2,7,8,9,10].

Previously, various regulatory factors involved in controlling the development of trichomes have been identified through deep studies of trichome growth and development [1,5,11,12]. MYB-bHLH-WD40 (MBW) acts as a trichome-formation complex and regulates trichome development in *Arabidopsis.* It consists of R2R3 MYB transcription factor GL1 (GLABROUS1) or MYB23, bHLH transcription factor GL3 (GLABRA3) or EGL3 (ENHANCER OF GLABRA3) and WD40 repeat protein TTG1 (TRANSPARENT TESTA GLABRA1) [1,3,5,13,14]. The MYB-bHLH-WD40 complex can bind to the promoter of HD-ZIP transcription factor GL2 (GLABRA2), which can activate the expression of *GL2* and induce trichome formation. GL2 is directly involved in regulating the growth and development of trichomes [15,16,17,18]. The transcription factors GIS (glabrous inflorescence stems), GIS2, GIS3, ZFP5 (zinc-finger protein 5), ZFP6 and ZFP8 encode C2H2 type zinc-finger proteins and control trichome development by functioning upstream of the trichome-formation complex MBW in *Arabidopsis* [19,20,21,22,23,24]. The functions of GIS and its subfamily members are partially redundant and complementary. In addition, various studies have reported the involvement of phytohormones in trichome formation via using their biosynthesis and signaling mutants [24,25,26,27,28,29]. Gibberellins (GA), as the most widely studied phytohormone, regulate the expression level of the GIS family and trichome-formation-complex genes via forming a gibberellin-responsive regulatory network to control trichome development in *Arabidopsis* [20,22,24].

TOE1 (TARGET OF EARLY ACTIVATION TAGGED 1) transcription factor belongs to the evolutionarily conservative AP2/ERF gene family [30,31,32,33]. The C-terminal of its amino acid sequence contains the conserved AP2 domain EAR motif, which can identify specific DNA cis-elements [34,35,36]. Previous studies have shown that *TOE1* transcripts are targeted and degraded by miR172, and TOE1 is involved in a variety of plant growth and development processes, including the transformation of growth-stage and stress responses [32,33,35,37,38]. Previous studies have found that TOE1/TOE2 are controlled by the miR156-SPL regulation module of developmental timing and influence flowering time in *Arabidopsis* [32,33]. Recent results have shown that TOE1 may regulate transcriptional repression of GL1 through histone deacetylation and influence trichome production on the abaxial (lower) surface of the rosette leaf in *Arabidopsis* [30]. However, the regulation pathway of abaxial trichome is different from that of the adaxial and main-stem inflorescence trichome [14,30,39]. Consequently, whether TOE1/TOE2 are involved in *Arabidopsis* trichome formation of the adaxial side and main-stem inflorescence is unclear. The purpose of this study was to investigate the function of TOE1/TOE2 in controlling trichome initiation in *Arabidopsis thaliana*. Our results showed that TOE1/TOE2 could interact with GIS gene family proteins and further regulate the expression of *GL1* and *GL3* to control the growth and development of the main-stem inflorescence trichome in *Arabidopsis*. These results will provide significant information on the function of TOE1/TOE2 in regulating trichome initiation in *Arabidopsis*.

## 2. Results

### 2.1. TOE1/TOE2 Are Involved in Main-Stem Inflorescence Trichome Initiation in Arabidopsis

**Figure 1:**
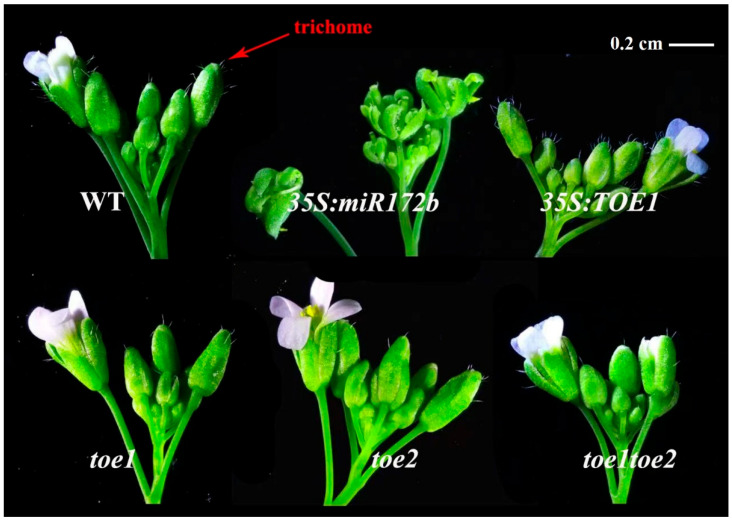
Phenotypes of main inflorescence stems of the *Arabidopsis thaliana*. Main inflorescence stems of the *Arabidopsis thaliana* WT, 35S:*miR172b* and 35S:*TOE1* above, main inflorescence stems of the *Arabidopsis thaliana toe1*, *toe2* and *toe1toe2* below. Main-stem inflorescence analyzed when the first flower opens. Bars = 0.2 cm.

**Figure 2:**
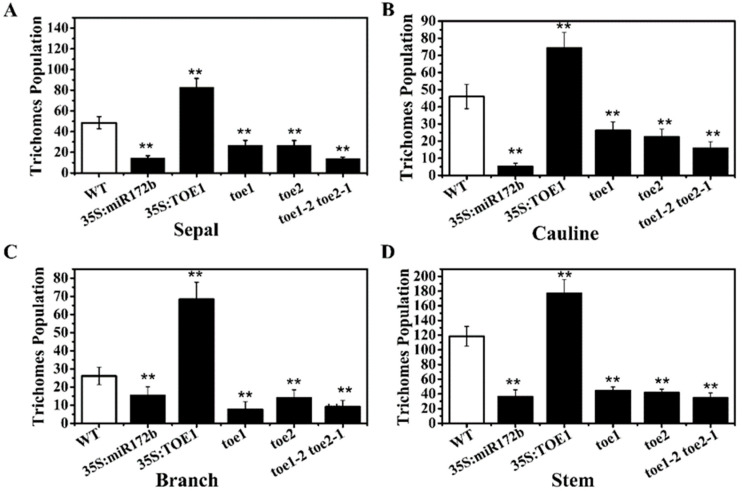
*Arabidopsis* trichomes in different lines. Trichome numbers on the sepals (**A**), the second cauline leaves (**B**), the second branches (**C**) and the main stems (**D**). Trichome number was the average number of counting at least 16 plants. Error bars indicate standard error. **, *p* < 0.01.

In order to explore the role of TOE1/TOE2 in *Arabidopsis* trichome, *Arabidopsis* wild type, *35S:miR172b* and *35S:TOE1* transgenic lines, *toe1*, *toe2* and *toe1toe2* mutants were used for this study. *35S:TOE1* is an overexpression transgenic line of *TOE1*, while *toe1*, *toe2* and *toe1toe2* are T-DNA insertion mutants. *35S:miR172b* is an overexpression transgenic line of *miR172b* which targets and degrades the mRNA transcripts of *TOE1* and *TOE2*. Our phenotypic observation results showed that the trichome production of sepals in the main-stem inflorescence of *35S:TOE1* was obviously higher than that of the wild type (Figure 1) and the trichome production of sepals in the main-stem inflorescence of *35S:miR172b*, *toe1*, *toe2* and *toe1toe2* was obviously lower than that of wild type. Moreover, *toe1toe2* had fewer trichomes than *toe1* and *toe2*. In addition, the defect phenotype of 35S:miR172b in the inflorescence trichome is most likely due to the downregulation of *TOE1* which is targeted by miR172b. To further verify these results, the numbers of *Arabidopsis* trichomes in the wild type and different lines were statistically analyzed. The results showed that *35S:TOE1* transgenic lines exhibited a much higher number of trichomes on the sepals, the second branch, the main stem and the cauline leaf than WT plants in the main-stem inflorescence. However, *35S:miR172b*, *toe1*, *toe2* and *toe1toe2* showed opposite phenotypes (Figure 2). These results are consistent with those shown in Figure 1. Collectively, these results suggested that miR172 and TOE1/TOE2 regulate the development of the trichome in the main-stem inflorescence of *Arabidopsis*.

### 2.2. TOE1/TOE2 Act Upstream of GL1 and GL3

**Figure 3:**
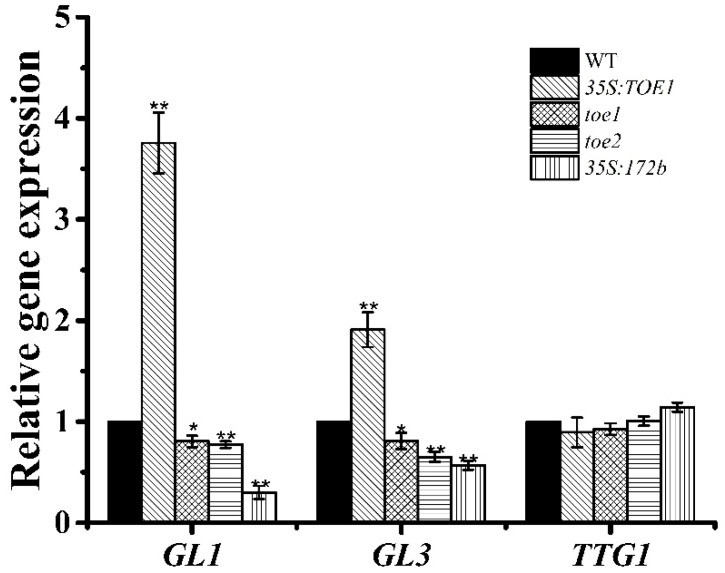
The expression on main stem of *GL1, GL3* and *TTG1* in the WT, *35S:TOE1, toe1*, *toe2* and *35S:miR172b*. Error bars represent SE. Student’s t-test was calculated at the probability of either 5% (*, *p* < 0.05) or 1% (**, *p* < 0.01).

The formation and development of trichomes is mainly regulated by trichome-formation complex MBW and zinc-finger protein transcription factors [3,5] In order to find the genetic position of *TOE1/TOE2* in the trichome initiation pathway, we examined the relative expression level of *TTG1*/*GL1*/*GL3* in WT, *35S:TOE1, toe1*, *toe2* and *35S:miR172b* transgenic lines. The results showed that the expression levels of *GL1* and *GL3* in the lines of *35S:TOE1* was significantly higher as compared to the wild type. In addition, the expression levels of *GL1* and *GL3* in the lines of *toe1*, *toe2* and *35S:miR172b* was significantly lower as compared to the wild type (Figure 3). These results clearly showed that *TOE1*/*TOE2* acting on the upstream of *GL3* and *GL1* leads to positive regulation of the expression of *GL3* and *GL1*, which promotes trichome formation and development in *Arabidopsis*.

### 2.3. TOE1/TOE2 Interact with GIS

**Figure 4:**
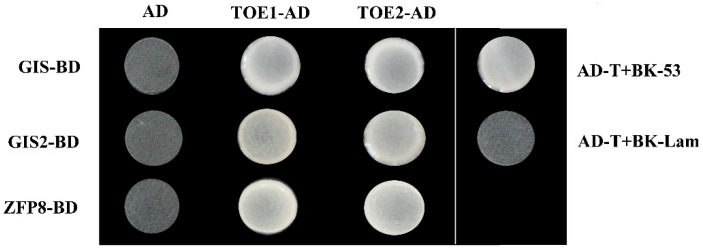
TOE1/TOE2 interact with GIS in a yeast two-hybrid assay. Culture results of yeast tetrahydric medium (SD/-Trp/-Leu/-His/-Ade). AD-T+BK-53 is the positive control and AD-T+BK-Lam is the negative control.

Previous research has proved that GIS family genes act on the upstream of trichome initiation complex TTG1/GL1/MYB23/GL3/EGL3 in *Arabidopsis* and participate in the regulation of trichome development [19,20,21,22,23,24]. In this study, the results showed that *TOE1*/*TOE2* positively regulate the expression level of *GL3* and *GL1* and promote trichome development by functioning upstream of *GL3* and *GL1*. Due to both TOE1/TOE2 and GIS family genes acting upstream of GL3 and GL1, we explored whether TOE1/TOE2 and GIS/GIS2/ZFP8 could physically interact to regulate the formation of trichome synergistically. For confirmation of this hypothesis, we performed the yeast two-hybrid experiment. The constructed vectors *TOE1/TOE2-AD* (pGADT7) and *GIS*/*GIS2/ZFP8-BD* (pGBKT7) were co-transformed into yeast AH109 strain and cultured on SD/-Trp/-Leu medium for 2 d. Twelve uniform single colonies were selected as replicates and transferred to SD/-Trp/-Leu/-His/-Ade medium. The normal single colony which grows on SD/-Trp/-Leu medium indicated that the two proteins directly interact. As shown in Figure 4, the single yeast colony co-transformed by *GIS/GIS2/ZFP8-BD* and *TOE1/TOE2-AD* could grow normally on the SD/-Trp/-Leu/-His/-Ade medium. Therefore, yeast two-hybrid assays confirmed the protein–protein interaction between TOE1/TOE2 and GIS.

**Figure 5:**
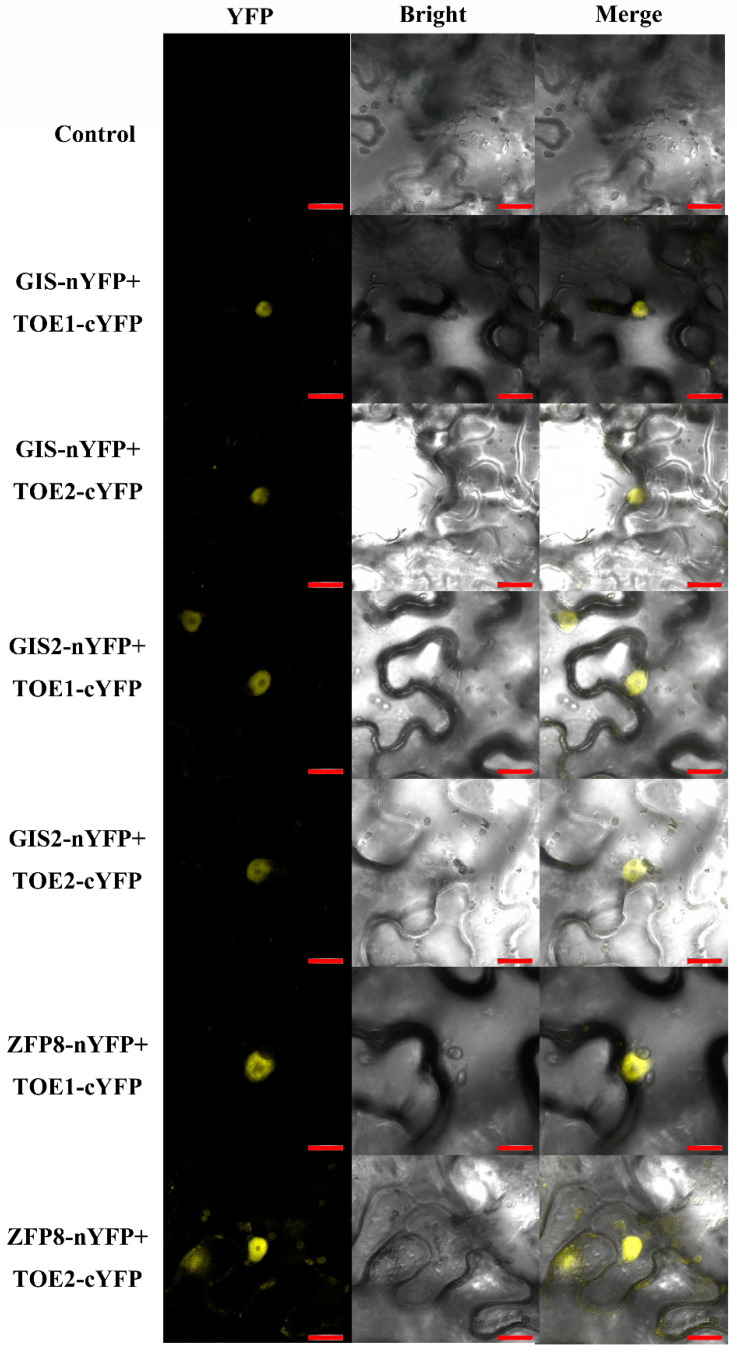
BiFC analysis of the interactions between TOE1/TOE2 and GIS in tobacco epidermal cells. Control is the negative control. Bars = 20 μm.

To further verify the interaction between TOE1/TOE2 and GIS/GIS2/ZFP8 protein, the constructs of *TOE1/TOE2-cYFP* and *GIS/GIS2/ZFP8-nYFP* were generated and transformed into Agrobacterium GV3101 for the bimolecular fluorescent complementation (BiFC) assay. By using the transient expression system of tobacco epidermal cells, the two Agrobacterium bacterial solutions of *TOE1/TOE2-cYFP* and *GIS/GIS2/ZFP8-nYFP* were mixed and injected into the tobacco epidermal cells. The YFP (yellow fluorescent protein) fluorescence was examined in the nuclei of tobacco epidermal cells when *TOE1/TOE2-cYFP* and *GIS/GIS2/ZFP8-nYFP* were co-expressed. The results showed that *TOE1/TOE2-cYFP* and *GIS/GIS2/ZFP8-nYFP* had fluorescence in the nucleus of tobacco epidermal cells (Figure 5). In contrast, the negative control did not show any YFP fluorescence signal. These YFP signals clearly verified the yeast two-hybrid results and further confirmed the interaction of TOE1/TOE2 with GIS/GIS2/ZFP8.

### 2.4. TOE1 Regulates Trichome Initiation through GA Signaling Pathway

**Figure 6:**
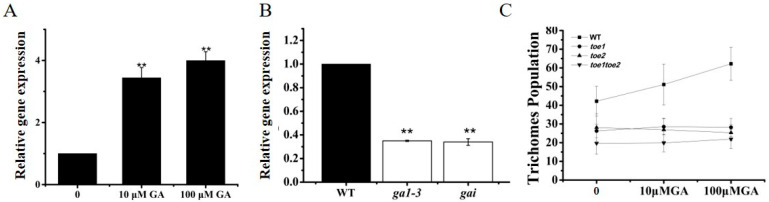
The Effects of GA on *TOE1* expression. (**A**) The relative expression of *TOE1* on main stems treated with different concentrations of GA (10 μM GA and 100 μM GA) and their corresponding negative control. (**B**) The relative expression of *TOE1* on the main stem of WT, *ga1-3* mutant and *gai* mutant. (**C**) Trichome population on the main stem of WT, *toe1* and *toe2* treated with different concentrations of GA (10 μM GA and 100 μM GA) and their corresponding negative control. Error bars represent SE. Student’s t-test was calculated at the probability of 1% (**, *p* < 0.01).

In *Arabidopsis*, GA plays a key role in trichome initiation by controlling the expressions of GIS family genes which function upstream of the MYB/bHLH/WD-repeat complex [14,40,41]. Our results indicated that TOE1/TOE2 interact with GIS/GIS2/ZFP8 function acting on the upstream of *GL3* and *GL1.* However, whether TOE1 is involved in the GA signaling pathway to regulate trichomes initiation is not yet explored. Therefore, for the confirmation of these pathways, we checked the expression level of *TOE1* on main stems treated with different concentrations of GA and their corresponding negative controls. The results showed that the expression of *TOE1* was significantly upregulated after GA_3_ treatment (Figure 6A). Moreover, the expression of *TOE1* was decreased significantly in the *ga1-3* and *gai* mutants as compared to WT (Figure 6B). For further clarification of these results, we used the exogenous GA hormone treatment approach. Twenty-day-old seedlings of WT, *toe1*, *toe2* and *toe1toe2* were sprayed with different concentrations of GA for the trichome phenotype observation experiment. The results showed that there was an increasing tendency of trichome production with the increasing concentration of GA in wild-type plants (Figure 6C). On the contrary, *toe1*, *toe2* and *toe1toe2* were insensitive to GA application (Figure 6B). These results indicated that *TOE1/TOE2* are required for GA signaling to regulate trichome initiation in the main-stem inflorescence in *Arabidopsis thaliana*.

## 3. Discussion

Trichome is regulated by a variety of genetic factors, including R2R3 MYB transcription factors, bHLH transcription factors, WD40 repeat proteins, HD-ZIP transcription factors, single repeat MYBs, C2H2 zinc-finger protein transcription factors, microRNA and ubiquitin/26S protease [3,5,14]. Previous research has found that miR156 directly targets SPL (squamosa promoter-binding protein-like), affects the mRNA level of SPLs and regulates the growth and development of trichomes in *Arabidopsis* [42]. In addition, SPL can promote the expression of miR172, while miR172 directly targets AP2-like transcription factors [31,33]. It has been reported that the miR156/SPL/miRNA172/AP2 regulatory model is involved in a variety of growth and development processes, including plant aging, flowering time, tuber formation, fruit growth and nodulation [32,33,35,37,38]. However, it is unclear whether miRNA172 and AP2-like transcription factors are involved in the regulation of trichome development in *Arabidopsis*. TOE1 which encodes a transcription factor belonging to the evolutionarily conserved AP2/ethylene responsive factor (ERF) plant family, plays a key role in flowering time, vegetative phase changes and stress response [32,33,35,37,38]. TOE1 physically interacting with KAN1, binds to the downstream of *GL1* and represses abaxial trichome initiation in *Arabidopsis* [30]. It is worth mentioning that the regulation pathway of adaxial trichomes is different from that of abaxial trichomes based on their presence and utility in *Arabidopsis*. *Arabidopsis* adaxial trichomes are mainly regulated by trichome activator complex MYB-bHLH-WD40 (MBW) [14,40,41]. In this study, we found that TOE1/TOE2 positively regulate the development of trichomes in the main-stem inflorescence, while miR172 has the opposite function (Figure 1 and Figure 2). Further experimental results showed that *TOE1*/*TOE2* act upstream of *GL3* and *GL1* and positively regulate the expression of *GL3* and *GL1* (Figure 3). These results indicate that miR172-*TOE1*/*TOE2* are involved in the main-stem inflorescence trichome initiation via regulating the expression of *GL3* and *GL1* in *Arabidopsis*. Similarly, C2H2 zinc-finger transcription factor GIS and its sub-family genes regulate trichome initiation through acting upstream of MBW trichome initiation complex in *Arabidopsis* [14,40,41]. However, whether there is an interaction between miR172-*TOE1*/*TOE2* and GIS family genes in trichome development regulation is still unknown. Our results indicate that TOE1/TOE2 physically interact with GIS, GIS2 and ZFP8, suggesting a novel mechanism for regulation of TOE1/TOE2-GIS upstream of MBW in trichome development regulation (Figure 4 and Figure 5).

GAs plays a crucial role in various plant growth and developmental regulatory pathway, such as floral induction, juvenile-to-adult transition, leaf expansion, hypocotyl and stem elongation and trichome initiation [14,40,41]. In *Arabidopsis*, the involvement of GA pathways in regulating trichome initiation is mainly by using pharmacological approach and GA-related gene mutants. External GA application promotes trichome initiation in the main-stem inflorescence in *Arabidopsis*. GA regulates trichome initiation accompanied by controlling key genes of trichome formation [1,5,11,12]. Previously, it has been reported that GA biosynthesis mutant *ga1-3* showed glabrous and stimulates earlier trichome initiation by GA application [41,43]. The GA-insensitive gai mutants is deficient in trichome production on stem inflorescence [41]. Significant low accumulation level of *GIS* family genes and *GL1* of inflorescence organs in *ga1-3* and gai mutants compared to wild-type plants [21,22,23,41,44]. In addition, the analyses of molecular, genetic and phenotype indicated that GIS family genes function upstream of trichome initiation complex and influence trichome initiation through GA signaling pathway in *Arabidopsis* [21,22,23,41,44]. However, miR156-targeted *SPL* regulating trichome development is independent of the GA-*GIS* pathway in *Arabidopsis* [45]. Our phenotype and genes expression data suggested that TOE1/TOE2 are required for GA signaling to regulate trichome initiation in the main-stem inflorescence (Figure 6). 

In addition, the same genetic factor may participate in a variety of growth and development regulatory pathway, and different plant growth and development processes also have interaction with each other. It is reported that flowering regulation and trichome formation share some phytohormones and transcription factors [14,39]. Previous study demonstrated that miR172-TOE1/TOE2 are involved in the plant phase transition and flowering time regulation [33]. Our results demonstrated that miR172-TOE1/TOE2 act upstream of MBW trichome-formation complex, interact with GIS, GIS2 and ZFP8 and regulate trichome initiation though GA signaling in *Arabidopsis.* These findings will increase our understanding of how flowering regulation and trichome initiation are intimately connected in plants. Further multifaceted biology approaches are expected to elucidate the molecular mechanism of the interaction between TOE1/TOE2 and GIS/GIS2/ZFP8 and the crosstalk between flowering regulation and trichome initiation in *Arabidopsis*.

## 4. Materials and Methods

### 4.1. Plant Materials and Treatments

All the transgenic lines and their corresponding wild-type seeds were surface-sterilized with chlorine gas for 4–5 h and then soaked in sterile water. The mutant lines *toe1* (At2g28550, Salk_069677) and *toe2* (At5g60120, Salk_065370) were obtained from the Arabidopsis Biological Resource Center (ABRC) [33]. In addition, the *ga1-3* (At2g01570, CS3104) and *gai* (At1g14920, CS63) mutants were obtained from the Nottingham Arabidopsis Stock Centre (NASC) [41]. Following with 4 °C treatment in the dark for 3–7 d, sterilized seeds were grown on Murashige and Skoog medium, then placed in a plant growth chamber under 16 h light at 22 °C and 8 h dark at 18 °C cycle. After 7–8 d, well-grown seedlings were potted in soil pots to continue growth [22,44,46,47].

### 4.2. RNA Extraction and Quantitative Real-Time PCR (qPCR)

Total RNA was extracted with Trizol reagent, then reverse-transcribed into cDNA strand by the M-MLV reverse transcriptase following the manufacturer’s protocol (Takara). Quantitative real-time PCR was carried out using a CFX96^TM^ Real-time Detection System according to SYBR Premix Ex TaqII (TaKaRa). The actin gene was used as a reference gene. Relative expression level of genes was calculated by the 2^−∆∆Ct^ formula [48,49]. Quantitative real-time PCR experiments were performed at least twice on three biological triplicates with similar results [50,51].

### 4.3. Gene Cloning and Vector Construction

The full-length CDS of *TOE1, TOE2*, *GIS*, *GIS2* and *ZFP8* were amplified by PCR using KOD FX (TOYOBO) and inserted into the *NotI* and *SalI* sites of the Gateway entry vector pENTR-1A vector (Invitrogen). Then, the ends of the insert from reconstructed pENTR-1A vectors were sequenced and subsequently recombined into the corresponding destination vector [52,53]. For the yeast two-hybridization experiment, the destination vectors pGBKT7 and pGADT7 were used for creating the constructs of *TOE1-AD, TOE2-AD*, *GIS-BD*, *GIS2-BD* and *ZFP8-BD*. P2YN and P2YC were employed to generate the constructs of *TOE1-cYFP, TOE2-cYFP*, *GIS-nYFP*, *GIS2-nYFP* and *ZFP8-nYFP* for the bimolecular fluorescent complementation analysis experiment. *Escherichia coli* DH5α was used for preserving the reconstructed vector. Yeast Strains AH109 and *Agrobacterium tumefaciens* strain GV3101 were used for yeast two-hybridization and BiFC analysis, respectively. 

### 4.4. Trichome Production Analysis

Trichomes on the sepals, main stems, branches and leaves at the similar positions were selected and measured in the same developmental stage. All sepals from the stem inflorescence were evaluated for counting trichomes. Main-stem trichome density was evaluated by counting trichomes on 2 cm of main stem length, 1.5 cm from the base of the main stem. Trichome number on branches was monitored by counting all trichomes on the second branches per plant. Total leaf trichome production on the second cauline leaves was measured for leaf trichome-number counting. For each of the treatment×genotype combinations, a minimum 16 plants were used for the statistical analysis of trichome production [22,23,44].

### 4.5. Yeast Two-Hybridization Assay

The reconstructed vectors pGBKT7 and pGADT7 were co-transformed into yeast strain AH109 following yeast strain AH109 competence transformation methods. pGADT7-*RecT*+pGBKT7-*Lam* and pGADT7-*RecT*+pGBKT7-*53* were used as a negative control (CK) and a positive control (CK+), respectively. The transformants were selected on synthetic dropout (SD) medium lacking leucine and tryptophan (-Leu and -Trp) in the dark at 30 °C for 48 h. The colonies on the positive and negative controls were then screened for growth on SD medium lacking adenine, histidine, leucine and tryptophan (–Ade, -His, -Leu and -Trp) at 30 °C in the dark [8,54].

### 4.6. Bimolecular Fluorescent Complementation (BiFC) Assay

All P2YN and P2YC constructed vectors were transformed into *Agrobacterium tumefaciens* strain GV3101 which was used for the infiltration of *Nicotiana benthamiana* leaves. Different combinations of constructs were mixed 1:1 and co-transformed into leaves of *Nicotiana benthamiana* (*Nicotiana benthamiana* plant grew for 4–6 weeks) and incubated in the dark at 23 °C for 24–72 h. Fluorescence in the lower epidermal cells of *Nicotiana benthamiana* leaves was examined and imaged using a fluorescence microscope (Nikon) [55,56,57].

### 4.7. GA Treatment

Plant hormone GA_3_ (Sigma) was used in external GA treatment experiments, as previously described. For gene expression analysis with GA treatment, *Arabidopsis* plants were sprayed with different concentrations (10 μM and 100 μM) of GA and negative control when the first five to six leaves had emerged. After 4 h treatment, a minimum of eight plants were harvested for RNA extraction. Twenty-day-old plants were sprayed twice a week with 10 μM GA_3_, 100 μM GA_3_ or their corresponding negative control to observe the trichome phenotype with GA treatment. The trichomes were then harvested for counting analysis when young inflorescence shoots of plants had come into the mature period [24,58].

### 4.8. Statistical Analysis

All data were tested by means of ANOVA for significance by using IBM SPSS Statistics. The significant difference between means was analyzed using Student’s t-test at the probability with 5% (*, *p* < 0.05 with significant level) or 1% (**, *p* < 0.01 with significant level), as previously described [20,23,24,46,58].

## Data Availability

Not applicable.
